# Economic burden of surgical site infections within the episode of care following joint replacement

**DOI:** 10.1186/s13018-019-1224-8

**Published:** 2019-06-27

**Authors:** Ayoade Adeyemi, Paul Trueman

**Affiliations:** 1 Smith & Nephew, Inc., 150 Minuteman Road, Andover, MA 01810 USA; 2 Smith & Nephew, Inc., Hull, UK

**Keywords:** Surgical site infection, Total joint arthroplasty, Economic, Burden

## Abstract

**Background:**

Recent policy initiatives, including Bundled Payments for Care Improvement (BPCI) Initiative by the Centers for Medicare and Medicaid Health Services (CMS), encourage healthcare providers to manage the total episode of care, rather than just the surgical episode. Surgical site infections (SSI) following total joint replacement result in preventable morbidity and suffering for patients and excess healthcare utilization for healthcare providers. This study sought to estimate the additional resources associated with SSIs within the 90-day episode of care following hip and knee joint replacement.

**Methods:**

Using the 2013 Nationwide Readmissions Database (NRD), healthcare resource utilization was compared between propensity score matched patient groups with and without SSI-related readmissions within the 90-day episode of care following total joint replacement.

**Results:**

Surgical site infections were associated with significantly longer hospital length of stay and increased costs following hip and knee joint replacement procedures. Generalized estimating equation regression results confirmed that additional costs associated with SSIs following both cohorts were significant, with additional hospital length of stay and costs following total hip and knee replacement procedures ranging from 4.9 to 5.2 days and $12,689 to $12,890, respectively.

**Conclusion:**

Surgical site infections following total joint replacement account for significant additional healthcare resource use within the 90-day episode of care.

## Introduction

As life expectancy continues to improve, healthcare administrators anticipate a corresponding rise in the incidence of total joint replacement, particularly primary hip and knee procedures [1–3]. The demand for total knee arthroplasty procedures has been projected to increase to about 3.5 million procedures annually by 2030, and a corresponding increase in Medicare payments to hospitals [4]. With an increasingly aging population presenting with comorbid conditions, managing the risk of postoperative complications, including surgical site infections (SSIs), represents a significant challenge to healthcare providers [5–7]. SSIs following total joint replacement are associated with significant healthcare utilization and worsened quality of life in patients [7, 8]. de Lissovoy et al. projected that by 2020, SSI-related readmissions following surgical procedures would account for almost one million additional inpatient days and $1.6 billion in costs [9]. Peel et al. also confirmed that SSIs are one of the top 2 major complications over the first 30 days following a knee or hip replacement procedure [10].

Recent policy initiatives, including the Comprehensive Care for Joint Replacement (CJR) program from the Centers for Medicare and Medicaid Health Services (CMS), are intended to encourage healthcare providers to consider best practices in managing patients over a 90-day episode of care, rather than just the acute care period [11, 12]. Bundled payments provide further financial incentives to put in steps to avoid postoperative complications that might result in excess treatment costs.

The current study sought to estimate the incidence and additional economic burden associated with surgical site infections within the 90-day episode of care period using the National Readmissions Database (NRD) of the Healthcare Cost and Utilization Project (HCUP). This unique database captures information on discharges with and without repeat hospital visits per year, allowing for the longitudinal study of patient populations through a 12-month period, such that readmissions and associated resource use following an event of interest can be captured and followed over time. With an increased emphasis on improved healthcare delivery while containing costs, this database has the capability of identifying causes of readmissions, making it possible to critically assess relevant patient populations and proactively develop strategies to successfully mitigate inefficiencies, while improving outcomes. The HCUP NRD is a publicly available (https://www.hcup-us.ahrq.gov/db/nation/nrd/nrddbdocumentation.jsp) national database that captures all-payer hospital inpatient-related events, and its flexibility allows various readmission-related analyses. Capturing more than 14 million discharges in 2013, 21 US states are accounted for in the database, representing over 2000 hospitals.

The present study was aimed at estimating the economic burden of SSI within 90 days following primary hip/knee joint replacement procedures.

## Methods

Using the 2013 NRD, discharges that were identified to be at least 45 years old with an International Classification of Diseases, Ninth Revision, Clinical Modification (ICD-9-CM) primary procedure code for an elective total hip or knee joint replacement procedure were included into the study. Patients who presented with bilateral joint procedures were excluded from the study.

The date of admission for the joint replacement was defined as the index date, and each patient was followed forward in time to capture any repeat visits associated with surgical site infections within 90 days of the primary joint replacement procedure. Readmissions due to a primary diagnosis of surgical site infections identified based on the presence of any of the ICD-9 CM diagnostic codes which include 99666, 99667, 99669, 99851, and 99859 were identified (see Table 1). Other discharge-level information extracted from the NRD includes age, gender, primary payer information, comorbidity measures, event dates, and healthcare utilization data (hospital length of stay, costs).

**Table 1:** Procedures/diagnoses by ICD-9 codes

Total joint replacement procedures	
Primary THA	81.51
Primary TKA	81.54
Surgical Site Infections ICD-9 CM diagnosis code	
Infection and inflammatory reaction due to internal joint prosthesis	99666
Infection and inflammatory reaction due to other internal orthopedic device, implant, and graft	99667
Infection and inflammatory reaction due to other internal prosthetic device, implant, and graft	99669
Infected postoperative seroma	99851
Other postoperative infection	99859

Total hospital costs were derived from total charges and cost-charge ratio data provided by HCUP, and these were estimated as the total costs associated with a patient from index hospital admission (defined as joint replacement) through the 90-day post-discharge period.

### Statistical analyses

Frequencies, means, and standard deviations were used in univariate analyses to describe the study population; thereafter, using a caliper-propensity score without replacement matching technique, patients identified to present with SSI within the 90-day post-index event discharge period were matched with patients without SSIs based on age, gender, and primary payer status (Medicare, Medicaid, private insurance, and the uninsured). Other baseline variables included were Zip income quartile, hospital bed size, hospital teaching status, and Charlson comorbidity scores (see Table 2 for details). Propensity scores (PS) were derived based on multinomial logistic regression; thereafter, study groups were matched based on a caliper setting of < 0.01. Baseline characteristics before and after the PS match were compared to assess whether balance was achieved between the matched groups. Following PS matching, paired *t* tests and generalized estimating equations which accounted for the matched nature of the final study sample were used to assess the relationship between hospital resource use and the presence/absence of SSIs following TJA. All analyses were carried out using the statistical analytical software, SAS 9.4. Descriptive statistics (mean, standard deviation, and frequency) were used to summarize baseline sociodemographic, clinical characteristics, and health care utilization cost patterns. McNemar’s and generalizing estimating equation (GEE) regression analyses were also conducted as deemed appropriate.

**Table 2:** Comparison of baseline characteristics by surgical site infection status pre- and post-match in the total hip arthroplasty cohort

		Pre-match		Post-match
	Total	No SSI	SSI	No SSI
*N*	48,143	47,740	403	403
Mean age (SD)	65.45 (10.87)	65.46 (10.87)	64.81 (10.99)	64.59 (10.86)
Sex (%)				
Male	22,000 (45.7)	21,822 (45.71)	178 (44.17)	189 (46.9)
Female	26,143 (54.3)	25,918 (54.29)	225 (55.83)	214 (53.1)
Payer (%)				
Medicare	26,264 (54.6)	26,029 (54.52)	235 (58.31)	231 (57.32)
Medicaid	1459 (3.03)	1432 (3)	27 (6.7)	30 (7.44)
Private insurance	18,504 (38.47)	18,376 (38.49)	128 (31.76)	130 (32.26)
Self-pay	315 (0.65)	314 (0.66)	1 (0.25)	2 (0.5)
No charge	61 (0.13)	59 (0.12)	2 (0.5)	2 (0.5)
Other	1502 (3.12)	1493 (3.13)	9 (2.23)	7 (1.74)
Unknown	38 (0.00)	37 (0.08)	1 (0.25)	1 (0.25)
Hospital bedsize (%)				
Small	7500 (15.58)	7444 (15.59)	56 (13.9)	52 (12.9)
Medium	12,482 (25.93)	12,380 (25.93)	102 (25.31)	96 (23.82)
Large	28,161 (58.49)	27,916 (58.48)	245 (60.79)	255 (63.28)
Zip income quartile (%)				
0–25th percentile	9457 (19.95)	9367 (19.62)	90 (22.33)	94 (23.33)
26th–50th percentile	12,301 (25.95)	12,192 (25.54)	109 (27.05)	107 (26.55)
51st–75th percentile	13,035 (27.5)	12,929 (27.08)	106 (26.3)	103 (25.56)
76th–100th percentile	12,604 (26.59)	12,512 (26.21)	92 (22.83)	95 (23.57)
Unknown	746 (0.00)	740 (1.55)	6 (1.49)	4 (0.99)
Hospital teaching status (%)				
Metropolitan non-teaching	22,070 (45.84)	21,863 (45.8)	207 (51.36)	200 (49.63)
Metropolitan teaching	21,865 (45.42)	21,707 (45.47)	158 (39.21)	166 (41.19)
Non-metropolitan teaching	4208 (8.74)	4170 (8.73)	38 (9.43)	37 (9.18)
Comorbidity Index (%)				
0–1	37,938 (78.8)	37,627 (78.82)	311 (77.17)	316 (78.41)
2–3	3873 (8.04)	3843 (8.05)	30 (7.44)	24 (5.96)
> 3	6332 (13.15)	6270 (13.13)	62 (15.38)	63 (15.63)

The top and bottom 1% of both cost and LOS data were winsorized in order to curtail the possible underestimation/overestimation effects of outliers on the study results.

### Outcomes

The main dependent and independent variables were total hospital costs and the presence/absence of a surgical site infection within 90 days following a total joint replacement respectively. The association between surgical site infections and cost and length of stay in the hospital were compared with non-SSI discharges following a 1:1 match in both adjusted and unadjusted analyses. The regression analyses employed took into account the positively skewed nature of healthcare resource use, assuming gamma and negative binomial distributions with log link function for costs and LOS respectively.

## Results

**Fig. 1:**
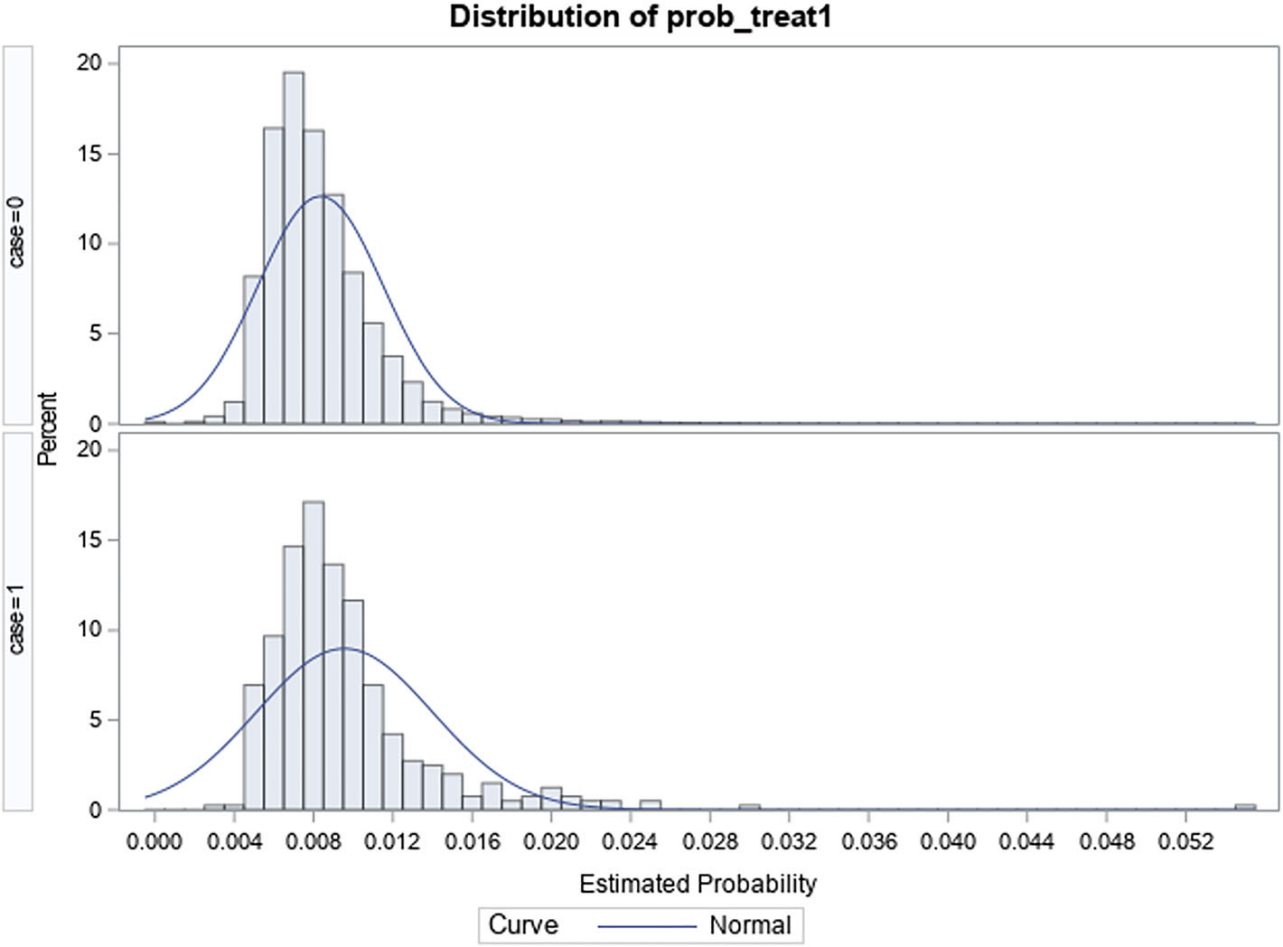
Graphic distribution of propensity scores after match in the total hip arthroplasty cohort

**Fig. 2:**
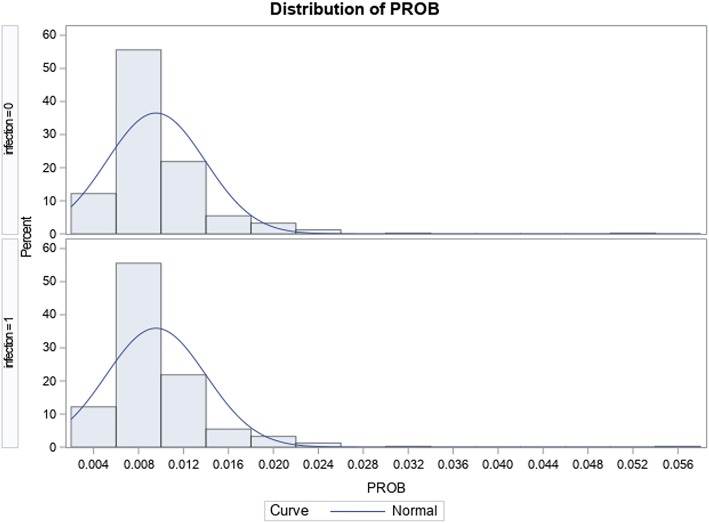
Graphic distribution of propensity scores before match in the total hip arthroplasty cohort

A total of 48,143 adult ≥ 45-year-old patients were identified to have met inclusion criteria within the study period; of this, 403 (0.84%) were identified to have surgical site infection-related readmissions. Table 2 shows how the SSI and non-SSI hip replacement cohorts compare before and after PS matching; the observed overlap between groups in Fig. 1 compared to Fig. 2 shows that balance was achieved between the 2 sub-populations after match. Table 3 shows the distribution of the specific SSI responsible for readmission, with “Infection and inflammatory reaction due to internal joint prosthesis” and “Other postoperative infection” accounting for 49.9% and 41.4% of surgical site infection-related readmissions respectively. In both unadjusted and adjusted analyses, Table 4 shows that the mean difference in total costs ($12,890; *p* < 0.001) and LOS (5.2; *p* < 0.0001) between patients with SSIs (total costs $29,288; LOS 8.25 days) and patients without SSIs (total costs $16,398; LOS 3.03 days) within 90 days post-discharge was statistically significant.

**Table 3:** Distribution of surgical site infections in the total hip arthroplasty cohort

Surgical site infection ICD-9 code	Description of infection	Frequency (%)
99666	Infection and inflammatory reaction due to internal joint prosthesis	201 (49.88)
99667	Infection and inflammatory reaction due to other internal orthopedic device, implant, and graft	12 (2.98)
99669	Infection and inflammatory reaction due to other internal prosthetic device, implant, and graft	2 (0.5)
99851	Infected postoperative seroma	21 (5.21)
99859	Other postoperative infection	167 (41.44)
Total		403 (100)

**Table 4:** Relationship between surgical site infection status and resource use in total hip arthroplasty patients

	Total	SSI	No SSI	Difference	Unadjusted	Adjusted
*N*	806	403	403	–		
Total cost of care [$] (SD)	22,843.08 (10342)	29,288 (9290)	16,398 (6679)	12,890.20 (10,922)	< .0001	< .0001
Length of stay [days] (SD)	5.64 (3.44)	8.25 (2.73)	3.03 (1.58)	5.22 (3.00)	< .0001	< .0001

**Fig. 3:**
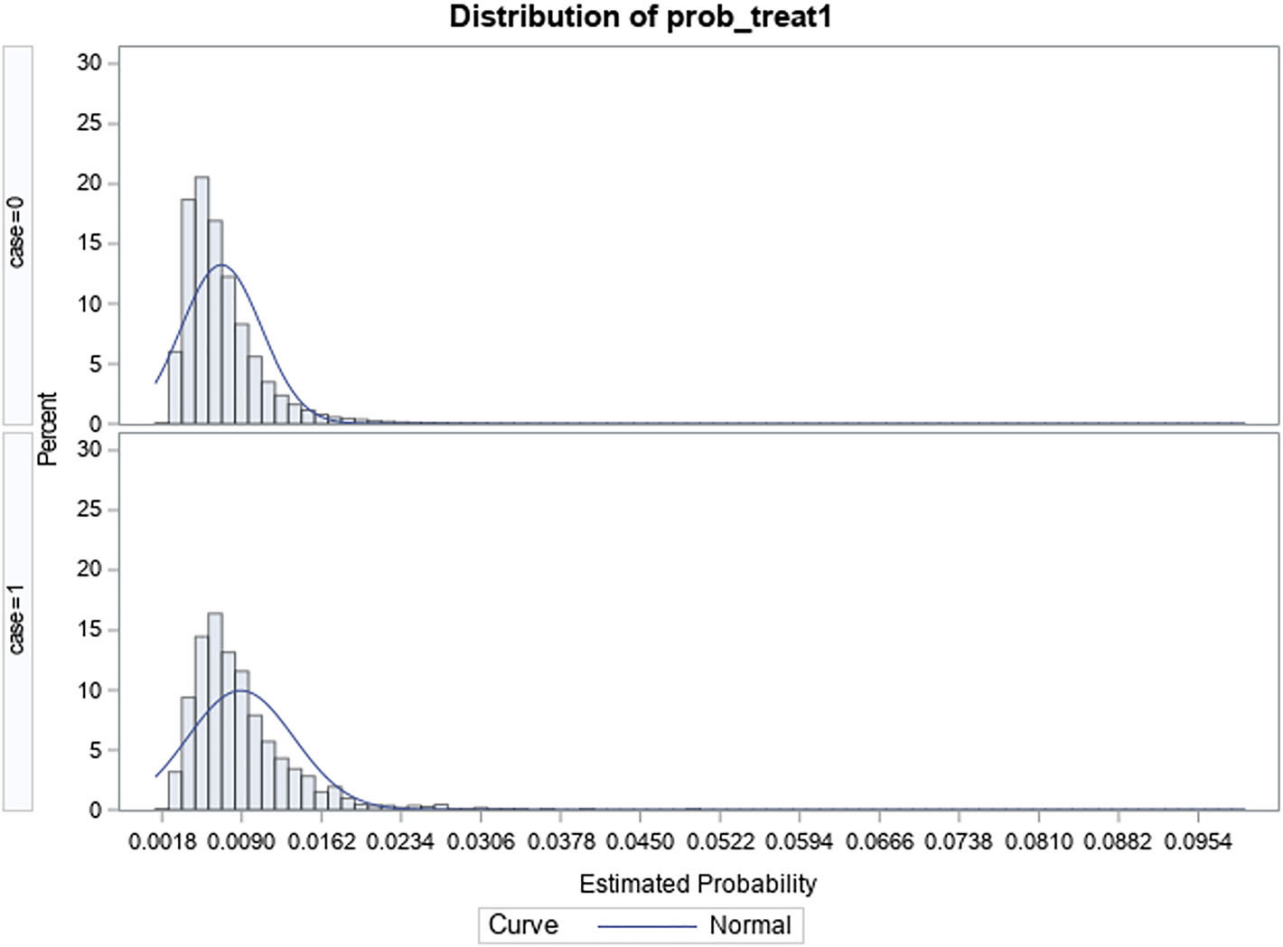
Graphic distribution of propensity scores after match in the total knee arthroplasty cohort

**Fig. 4:**
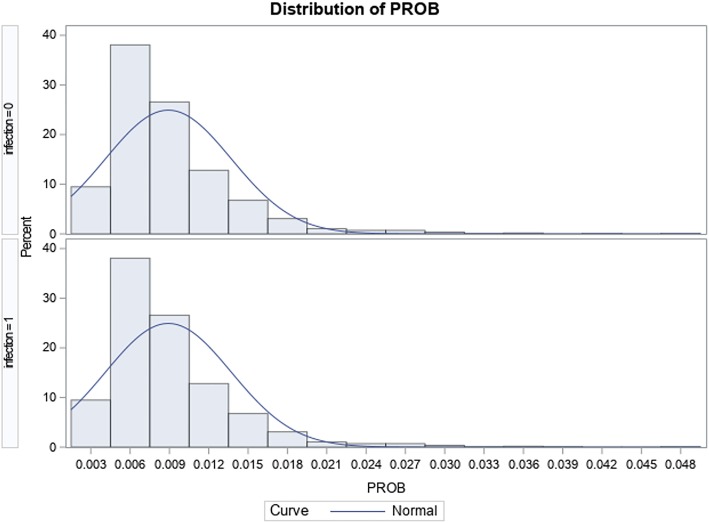
Graphic distribution of propensity scores before match in the total knee arthroplasty cohort

In the total knee replacement cohort, a total of 158,516 patients ≥ 45 years met inclusion criteria; of this, 1140 (0.72%) met the inclusion criteria for readmission due to surgical site infection within the 90-day episode of care period. Table 5 shows how the groups in the knee cohort differ before and after match; moreover, the overlap observed between groups in Fig. 3 confirms that balance was achieved between groups after the match when compared to before the match (see Fig. 4). Table 6 shows that the most common SSIs responsible for a readmission were “Infection and inflammatory reaction due to internal joint prosthesis” and “Other postoperative infection”, accounting for 49.8% and 46.1% respectively. There was a statistically significant additional cost of $12,689; *p* < 0.0001 associated with patients with SSIs ($28,576) compared to their peers without SSIs ($15,887); by LOS, patients readmitted due to SSIs (8.02 days) spent an additional 4.9 days; *p* < 0.0001 in the hospital compared to their peers without SSI-related readmissions with an LOS of 3.12 days (see Table 7).

**Table 5:** Comparison of baseline characteristics by surgical site infection status pre- and post-match in the total knee arthroplasty cohort

		Pre-match		Post-match
	Total	No SSI	SSI	no SSI
*N*	158,516	157,376	1140	1140
Mean age (SD)	66.25 (9.7)	66.3 (9.7)	65.3 (10.0)	65.8 (9.7)
Sex (%)				
Male	61,012 (38.5)	60,456 (38.4)	556 (48.8)	562 (49.3)
Female	97,504 (61.5)	96,920 (61.6)	584 (51.2)	578 (50.7)
Payer (%)				
Medicare	90,700 (57.2)	90,040 (57.2)	660 (57.9)	672 (59.0)
Medicaid	4607 (2.9)	4535 (2.9)	72 (6.3)	69 (6.1)
Private insurance	55,590 (35.1)	55,243 (35.1)	347 (30.4)	349 (30.6)
Self-pay	636 (0.4)	626 (0.4)	10 (0.9)	15 (1.3)
No charge	166 (0.1)	163 (0.1)	3 (0.3)	4 (0.4)
Other	6632 (4.2)	6585 (4.2)	47 (4.1)	31 (2.7)
Unknown	185 (0.1)	184 (0.1)	1 (0.1)	0(0)
Hospital bedsize (%)				
Small	28,404 (17.9)	28,223 (17.9)	181 (15.9)	172 (15.1)
Medium	42,309 (26.7)	41,997 (26.7)	312 (27.4)	298 (26.1)
Large	87,803 (55.4)	87,156 (55.4)	647 (56.8)	670 (58.8)
Zip income quartile (%)				
0–25th percentile	33,248 (21.0)	32,992 (21.0)	256 (22.5)	268 (23.5)
26th–50th percentile	42,535 (26.8)	42,237 (26.8)	298 (26.1)	286 (25.1)
51st–75th percentile	43,118 (27.2)	42,810 (27.2)	308 (27.0)	312 (27.4)
76th–100th percentile	37,061 (23.4)	36,799 (23.4)	262 (23.0)	264 (23.2)
Unknown	2554 (1.6)	2538 (1.6)	16 (1.4)	10 (0.9)
Hospital teaching status (%)				
Metropolitan non-teaching	78,155 (49.3)	77,586 (49.3)	569 (49.9)	581 (51.0)
Metropolitan teaching	64,844 (40.9)	64,391 (40.9)	453 (39.7)	461 (40.4)
Non-metropolitan teaching	15,517 (9.8)	15,399 (9.8)	118 (10.4)	98 (8.6)
Comorbidity Index (%)				
0–1	124,721 (78.7)	123,890 (78.7)	831 (72.9)	833 (73.1)
2–3	12,378 (7.8)	12,273 (7.8)	105 (9.2)	100 (8.8)
> 3	21,417 (13.5)	21,213 (13.5)	204 (17.9)	207 (18.2)

**Table 6:** Distribution of surgical site infections in the total knee arthroplasty cohort

Surgical site infection ICD 9 code	Description of infection	Frequency (%)
99666	Infection and inflammatory reaction due to internal joint prosthesis	568 (49.8)
99667	Infection and inflammatory reaction due to other internal orthopedic device, implant, and graft	41 (3.6)
99669	Infection and inflammatory reaction due to other internal prosthetic device, implant, and graft	3 (0.3)
99851	Infected postoperative seroma	3 (0.3)
99859	Other postoperative infection	525 (46.1)
Total		568 (49.8)

**Table 7:** Relationship between surgical site infection status and resource use in total knee arthroplasty patients

	Total	SSI	No SSI	Difference	*p* value	
					Unadjusted	Adjusted
*N*	2240	1120	1120	–	–	–
Total cost of care [$] (SD)	22,231.58 (10,762)	28,576.02 (10,468)	15,887.14 (6450)	12,689 (11,898)	< .0001	< .0001
Length of stay [days] (SD)	5.57 (3.4)	8.02 (3.0)	3.12 (1.7)	4.90 (3.5)	< .0001	< .0001

## Discussion

Although 400,000 hip and knee joint replacement procedures were completed in 2014, accounting for over $7 billion in hospitalizations [11], quality of care and costs still vary widely and surgical site infections (SSIs) continue to pose a significant burden to patients and the healthcare system. The recent emphasis on the 90-day episode of care bundled payment for the CJR program [11, 13] coupled with financial incentives, as part of the Bundled Payments for Care Improvement (BPCI) Initiative by CMS, make it imperative that healthcare providers understand both the clinical and financial implications of SSIs in the post-acute care period.

The findings of the current study confirm that the additional costs associated with SSIs within the 90-day episode of care following total joint replacement are significant. However, patient suffering due to additional treatments of these preventable infections are unquantifiable and so is the pressure that these events can put on providers that are subject to working based on bundled payments. With projections suggesting that joint replacement procedures are on the rise [14], there is a need for healthcare providers to take a more active and pragmatic approach to prevent complications, such as SSIs. This has the potential to reduce avoidable morbidity and associated mortality, improve patient satisfaction and quality of life, and contribute to improved clinical and financial performance.

Although there are treatment protocols and guidelines designed to improve perioperative efficiencies, curtail and/or prevent the incidence and severity of preventable surgical site infections following joint replacement [14–16], a more pro-active, individualized approach may be required to adequately address this issue. Surgical site infection prevention measures may involve the identification of high-risk patients and the proactive implementation of pre- and postoperative protocols to mitigate such preventable complications [17]. There is evidence supporting the prophylactic use of systemic antibiotics in the perioperative period; however, the use of antibiotic-loaded bone cement in reducing infections following joint replacement is inconclusive [14–16]. Other published studies have also reported the impact of a number of evidence-based protocols that have proven effective in the reduction of complications, such as the targeted use of negative pressure wound therapy (NPWT) on surgical incisions [15, 16, 18, 19]. Although the incidence of SSI within 90 days following total joint replacement from the present study is less than 1%, the associated resources used are significant especially as it adds to an already expensive procedure. Although other studies have reported incidence rates ranging from 0.3 to 2.5% [20–22], the risks of infection following hip and knee replacement are expected to rise to 6.5% and 6.8% respectively by 2030 [14], unless mechanisms are more proactively developed and executed to mitigate this projected increase.

With the surgical site infection-related readmissions observed to be primarily due to the internal device used, it would also be important to consider how outcomes differ by the specific internal joint prosthesis used in joint replacement. While device costs remain a significant determining factor in the choice of the prosthesis used, there is a need to assess how long-term outcomes impact overall treatment costs. The extended period of antibiotic treatment and revisions as a result of infections from surgical implants are considered significant and in most cases avoidable. This is of crucial financial importance as complication of device, implant, or graft is considered one of the five most expensive conditions by payer, accounting for over $12.4 million and 632,000 hospital stays [23].

Our study adds to the existing literature estimating the economic burden of SSIs over the 90-day episode of care period following hip and knee joint replacement, utilizing the Nationwide Readmissions Database, a nationally representative database with the capability of capturing readmissions following an event of interest [24–26]. We estimated the additional costs associated with surgical site infections following total joint replacement by applying a propensity score matching technique to account for possible bias between groups due to differences in baseline characteristics. We also focused on only readmissions that identified surgical site infections as the primary cause of readmission. The present study ensured that baseline covariates identified to be significant risk factors for infections following joint replacement procedures were comparable between the groups with and without SSIs. Some limitations of the study include a possible underestimation of the economic burden of surgical site infections due to the omission of surgical site infection-related readmissions considered as a secondary reason for readmission and outpatient-based treatments. Such events will be considered less severe, hence did not warrant a readmission. Another limitation, common with observational studies, is the inability to control for unmeasured covariates; however, our ability to adequately match all measured baseline covariates minimizes this effect. Furthermore, while the database contains about 17 million discharges per year, accounting for 27 states in the USA, the observed findings may not be generalizable beyond the population studied.

## Conclusion

Surgical site infections following total joint replacement are associated with significant healthcare costs within the 90-day episode of care. Healthcare providers, particularly those subject to quality improvement initiatives and bundled payments, are encouraged to consider evidence-based protocols to avoid excess morbidity, patient suffering, and the financial costs associated with these events.

## Data Availability

The data that support the findings of this study are available from The National Readmissions Database of the Healthcare Costs and Utilization Project, but restrictions apply to the availability of these data, which were used under license for the current study, and so are not publicly available. Data are however available from the authors upon reasonable request and with permission of The National Readmissions Database of the Healthcare Costs and Utilization Project.

## Abbreviations

BPCI: Bundled Payments for Care Improvement
CJR: Comprehensive Care for Joint Replacement
GEE: Generalizing estimating equation
HCUP: Healthcare Cost and Utilization Project
ICD-9-CM: International Classification of Diseases, Ninth Revision, Clinical Modification
LOS: Length of stay
NPWT: Negative pressure wound therapy
NRD: National Readmissions Database
PS: Propensity score
SSI: Surgical site infection
TJA: Total joint arthroplasty
